# Cross cultural adaptation and validation of audiovisual educational material for use in indigenous patients with rheumatoid arthritis

**DOI:** 10.1016/j.pecinn.2024.100363

**Published:** 2024-12-18

**Authors:** Joana Aguilar-Castillo, Ingris Peláez-Ballestas, José-Luis Montiel-Hernández, Cairo Toledano-Jaimes, Mario-Alberto Garza-Elizondo, David Zepeda-González, Diana-Lizbeth Gómez-Galicia

**Affiliations:** a Laboratory of Pharmaceutical Epidemiology, Facultad de Farmacia, Universidad Autónoma del Estado de Morelos, Cuernavaca, Morelos, México; b Rheumatology Unit, Hospital General de México "Dr. Eduardo Liceaga", México city, México; c Laboratory of Cytokines and Autoimmunity, Facultad de Farmacia, Universidad Autónoma del Estado de Morelos, Cuernavaca, Morelos, México; d Rheumatology Service, Hospital Universitario "Dr. José Eleuterio González", Universidad Autónoma de Nuevo León, Monterrey, México; e Clínica Esquipulas, San Cristóbal de las Casas, Chiapas, México

**Keywords:** Patient health education, Audiovisual educational material, Rheumatology, Pharmaceutical intervention, Indigenous patients

## Abstract

**Background:**

Culturally appropriate educational materials are necessary to improve health literacy among Indigenous populations. However, practically no such materials have been cross-culturally adapted and validated for Indigenous peoples based on compliance with efficacy components.

**Objective:**

To perform a cross-cultural adaptation and validation of audiovisual educational materials for adult patients with rheumatoid arthritis belonging to Indigenous communities in Chiapas, Mexico.

**Methods:**

Mixed-methods study consisting of three phases: 1) Spanish–Tzotzil translation and cross-cultural adaptation of seven previously designed and validated audiovisual educational materials; 2) qualitative validation; and 3) quantitative validation based on the efficacy components (attraction, understanding, induction to action, involvement, and acceptance). The information collected during the validation phases was recorded and transcribed for content analysis.

**Results:**

A total of 31 patients with rheumatoid arthritis participated in the study. Patients had a mean age of 49 years, ≥5 years since disease onset, low adherence to pharmacological treatment (<20%), and a high level of illiteracy (>80%). After three versions of the educational material, where elements of cultural identification were added, the efficacy components increased significantly to reach scores higher than 90%. This suggests that culturally-adapted materials could promote greater patient participation in treatment.

**Conclusion:**

This study shows the importance of cross-cultural adaptation in the design and validation of audiovisual educational materials for Indigenous populations; this aspect should be considered when implementing educational strategies for patients with chronic diseases.

**Innovation:**

First educational audiovisual material translated and adapted from Spanish to Tzotzil, with a cultural sensitivity approach to achieve educational goals and improve therapeutic adherence.

## Highlights

• There are few audiovisual materials for Indigenous people that meet the components of efficacy, which limits the cultural sensitivity necessary to offer successful health education among different linguistic and cultural groups.
• Cross-cultural adaptation and validation of audiovisual educational material is sufficient to improve compliance with the components of efficacy.
• Cross-culturally educational material in Tzotzil language for indigenous patients with rheumatoid arthritis allows them to understand their disease and treatments.
• Indigenous patients prefer that the audiovisual educational material includes photographs of real people, wearing their traditional clothing and carrying out everyday activities.
• Indigenous patients believe that the educational materials in this study are attractive, easy to understand, with recommendations that are easy to identify and carry out, do not contain offensive elements, and are aimed at people like them.

## 1 Introduction

Low therapeutic adherence in patients with rheumatoid arthritis (RA) is associated with more disease flares, increased disease activity, and high functional disability [1]. One of the factors associated with low therapeutic adherence is low health literacy [[1], [2], [3], [4]], usually among older adults, minority groups, people with limited resources, and those with less than 9 years of continuous schooling [5]. Additionally, patients often receive treatment instructions with overly technical information that hinders their understanding [6]. Therefore, the implementation of educational strategies that are understandable and acceptable from the perspective of patients is necessary to improve health care.

Previous studies have shown that audiovisual materials for patient education are more attractive and better received than other media; additionally, they can be used by anyone, regardless of social status or educational level [[6], [7], [8], [9]]. However, only 11% of the web accessible audiovisual educational materials in the field have adequate quality and clinical value [9]. Furthermore, it is difficult to ascertain if patient perspectives are being considered in these materials. For this reason, our research group has previously designed educational audiovisual materials on seven relevant topics for patients with RA [8]. These considered the input of specialists and patients, and were validated to ensure adequacy, appropriateness, approval and significance from the patients’ perspective. The validation of these materials was performed in line with the guide proposed by the United Nations Children’s Fund (UNICEF) [10], which proposes that the efficacy of educational materials must surpass 70% compliance in five different components: attraction, understanding, induction to action, involvement, and acceptance [8].

In Mexico, 22.3 million people who self-identify as Indigenous live in conditions of clear social disadvantage considering factors such as illiteracy, poverty, inequality and inequity [11]. These elements are not only associated with a low level of therapeutic adherence [5], but negatively impact health outcomes measures such as better care and health literacy [11,12]. Chiapas is the state with the second highest percentage of Indigenous population in all Mexico; for every 100 inhabitants, 28 self-identify as belonging to an Indigenous community [11]. Additionally, there is a wide ethnic diversity: Tzotzil (34.5%), Tzeltal (38.2%) and Ch’ol (15.9%) are the most numerous Indigenous groups in the region [13].

However, very few educational and audiovisual resources have been adapted to Indigenous sociocultural conditions and health care preferences [[16], [17]], limiting the understanding and acceptability of therapeutic interventions. Therefore, the development of such culturally adapted and validated educational audiovisual material [8] is necessary to reduce the care inequities for patients with RA, and improve health literacy and therapeutic adherence [6,14,15], especially for vulnerable populations such as Indigenous communities.

## 2 Materials and methods

### 2.1 Study design

**Fig. 1:**
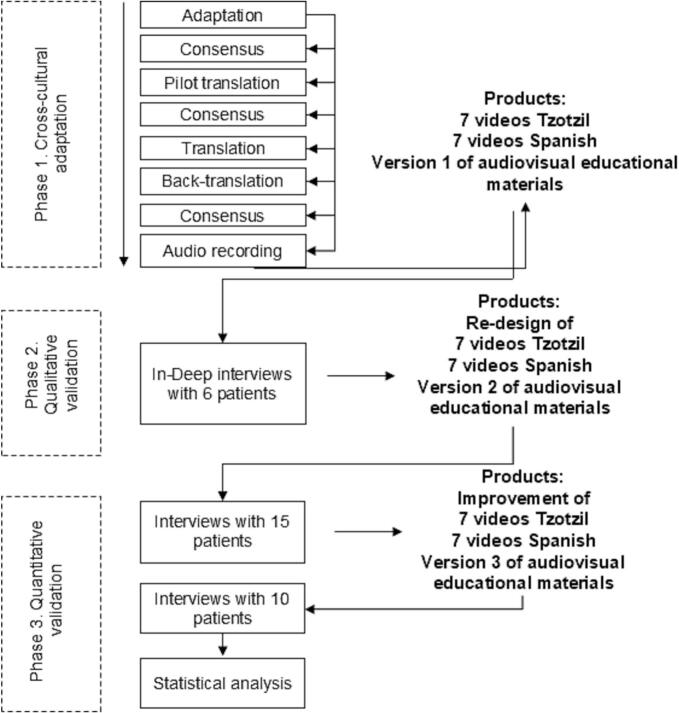
Diagram of the sequential mixed methodology to cross-culturally adapt and validate audiovisual educational materials for Indigenous patients. Phase 1: cross-cultural adaptation. Phase 2: sequential correction of audiovisual materials and qualitative validation. Phase 3: quantitative validation of audiovisual materials according to the efficacy components.

The Spanish-language educational audiovisual material that served as the basis of this study has been previously described [8]. It was developed with the collaboration of specialists and patients, determining that the information required by patients with RA is divided into seven topics. In this present study to cross-culturally adapt and validate the materials for Indigenous patients with RA, we followed a sequential mixed methodology in three phases, including qualitative and quantitative methods (Fig. 1). The qualitative methodology was employed to identify the patients’ opinions about the audiovisual educational material, considering the efficacy components. The quantitative methodology allowed us to quantify compliance in the target population considering the efficacy components.

#### 2.1.1 Audiovisual material for patients with rheumatoid arthritis

Seven audiovisual resources were produced in Mexican Spanish, with lengths ranging from 2:53 minutes to 3:54 minutes. Content includes patient’s concerns, doubts and beliefs, validated by assessing compliance with the five efficacy components proposed by UNICEF [10]. The original audiovisual materials in Spanish achieved 96% compliance according to patients, and 86.9% according to specialists (rheumatologists, pharmacists and medical anthropologists). The materials were presented by a pharmacist and simultaneously translated into the local language by Indigenous interpreters.

#### 2.1.2 Outcomes

The primary outcome of the study was to design audiovisual educational materials with a high level of compliance in the five efficacy components: attraction, understanding, induction to action, involvement and acceptance. The secondary outcome was to evaluate the impact of cross-cultural adaptation to achieve high levels of compliance with the efficacy components.

### 2.2 Patients

**Fig. 2:**
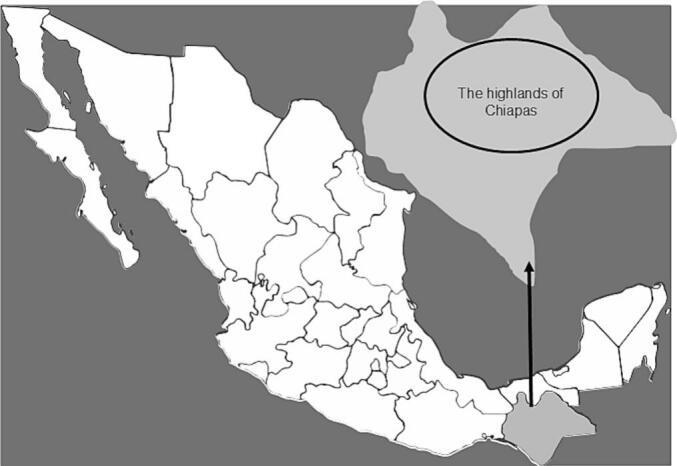
Map of Mexico and the highlands of Chiapas.

This present study included adult patients belonging to the three main Indigenous communities of the Chiapas highlands region (Fig. 2). Convenience sampling was used to select 31 patients who self-identified as Indigenous. Monolingual Tzotzil patients and bilingual patients (Spanish and Tzotzil, Tzeltal or Ch’ol) participated in the different stages of validation. All patients fulfilled the 2010 American College of Rheumatology (ACR)/European League Against Rheumatism (EULAR) criteria for RA classification. Patients were recruited to the Esquipulas Clinic, a primary healthcare center specializing in Indigenous care in the region, located in San Cristóbal de las Casas, Chiapas (Fig. 2). The patients were recruited during their routine rheumatology consultation.

### 2.3 Ethical considerations

The protocol with registration number FM/CEI/002/2019 was approved by the Ethics and Research Committees of the Hospital General de Cuernavaca “José G. Parres” (HGP/CEI/522/2018). The study conformed to the principles in the Declaration of Helsinki [18], and all data were treated in accordance with existing regulations to protect patient privacy. Informed consent was explained by a native interpreter to monolingual patients, and all patients expressed their consent through signature or thumb-print on the form depending on their preference.

### 2.4 Phase 1: Cross-cultural adaptation

The previously reported [8] seven audiovisual educational materials, along with the validation guide, were translated only into the local Indigenous Tzotzil language by three bilingual translators. Primary cross-cultural adaptation was done by consensus techniques during meetings between the local translators and J.A.C. to resolve inconsistencies. The secondary cross-cultural adaptation process [19] was carried out with the following steps: 1) translation of the audiovisual educational material scripts and the validation guide into Tzotzil in order to identify the content that could not be translated, that was offensive and/or was difficult to understand; 2) consensus meeting, translation (Tzotzil–Spanish) and retro-translation (Tzotzil–Spanish) of audiovisual educational material scripts and validation guide; 3) consensus meeting to assess understanding, writing, use of colloquial language, the equivalence of meaning (semantics) and concepts of each translation. This phase ended with recording the audio in Tzotzil language by a native speaker (E.V.P.); this voice-over translation was added to the seven audiovisual educational materials (see Fig. 1).

### 2.5 Phases 2 and 3: Validation using a qualitative and quantitative approach

Semi-structured and in-depth interviews [20] were conducted with patients with RA who attended the consultation during the period from September to October 2019. The previously adapted validation guide was used for the interviews. This guide contains questions aimed at identifying compliance with the efficacy components. The attraction component was assessed with the following questions: “Was the video too short, too long, or an adequate length?”, “Did you like the video?”, “Did you like the images?”, and “Did you like the colors?”. The understanding component was assessed with the question “Did you understand the information in the video?”. Acceptance was assessed with the question “Is there a word or image that makes you feel upset, offended or angry?”. Involvement was assessed by asking “Who do you think this video is for?”. Finally, induction to action was assessed with the question “Is this video asking you to do something?”

Patient interviews were voluntary and individualized, providing a comfortable environment and aided by three native Tzotzil bilingual interpreters. Both the qualitative and quantitative phases consisted of reading and explaining the informed consent (20 min. Approx.), administering the general data questionnaire (20 min. Approx.) and screening the audiovisual educational materials. The information collected during the interviews was first recorded and transcribed for the content analysis [21], which consists of classifying the responses to the validation guide as positive and negative, in order to identify opportunities for improvement of the audiovisual educational materials with the support of an interdisciplinary group of pharmacists, an immunologist and medical anthropologist. Additionally, validation using a quantitative approach was performed by evaluating the answers to the validation guide using a spreadsheet, in which responses were categorized with “1” for positive responses and “0” for negative responses. The compliance with the efficacy components was then calculated as the percentage of positive responses for each component. Importantly, the translators and the researcher made observations in a field diary during both the qualitative and quantitative phases, with the aim of improving the validation process and enhancing patient learning, by taking into account aspects that could influence the quality of the information obtained. An adapted Morisky-Green tool [22] was used to evaluate patient adherence to treatment with Disease Modifying Antirheumatic Drugs (DMARDs) during the qualitative and quantitative.

## 3 Results

### 3.1 Phase 1: Cross-cultural adaptation

The entire process is outlined in Fig. 1. An audiovisual educational material in Tzotzil was generated based on a previously validated version in Spanish. After reviewing the first translation, a total of 23 concepts were changed according to their semantic equivalence, since they do not exist in the Tzotzil language. Some of the modified concepts were: “rheumatoid arthritis” changed to “bone pain”, “disease in remission” to “non-active disease”, “pharmaceutical” to “the one who monitors medicines”, “health professionals” to “those who have medical study”, and “herbal” to “remedies”.

### 3.2 Phase 2: Qualitative validation

The first version of the audiovisual educational material was qualitatively validated by 6 Tzotzil monolingual patients. The interviews lasted an average of 59 minutes (52–63 minutes) depending on the number of questions and the patient’s understanding. All the participants were women living in a rural area, with a mean age of 58 years (44–74 years), and where the majority (66.7%) had RA for over 7 years. Additionally, two-thirds of the participants declared using medicinal plants and 83.3% were illiterate. Only half the patients were adherent to treatment; reasons for non-adherence to DMARDs were forgetfulness (66.66%), suspending treatment because they felt good (66.66%), not following a schedule (33.33%), and discomfort related to drugs (33.33%).

Patients asked a total of 20 questions during the interviews, which were answered by the pharmacist through an educational intervention. Half of the questions were related to diet (e.g. whether they could include consume meat or if hot foods/beverages could increase joint pain), and one fourth were about medications (e.g. whether they could take their medications with tea). To address these areas of concern and increase the relevance of the audiovisual educational materials for Indigenous patients, some images related to food, day-to-day activities like work, and the use of herbs were selected for inclusion. At this stage we also decided to shorten the audiovisual educational materials, which were repetitive, as well as decrease the brightness of the computer screen when sharing them with patients, who seemed tired.

When evaluating the efficacy components, the attraction component was fulfilled since patients liked elements of the audiovisual educational materials, such as voice, music, background, duration, colors and images. Acceptance was also fulfilled, since there were no reports of images or words being offensive. Regarding the involvement component, patients did not feel identified with the audiovisual educational materials; they believed the materials had been designed for doctors or for other patients instead. The understanding of the images was not satisfactory either (42.9%); patients attributed this to the color choices making them difficult to distinguish. On the other hand, patients stated that the audiovisual educational material enhances understanding of the disease (“I can explain my illness to myself”), of the importance of taking the medication according to the doctor’s instructions (“That I comply with what the doctor says and that I don’t suspend the medicine, thus healing and relieving the pain I have”), of the importance of communication with the doctor (“Tell doctors where the pain is”), among others (Table 1). Finally, the induction to action component was fulfilled since, according to the patient’s comments, the audiovisual educational materials have clear and easy to identify recommendations such as “Comply with the doctor’s recommendations”, “Don’t take medicines at the same time”, and “Properly store medications”, among others.

**Table 1:** Qualitative evaluation results corresponding to the components of understanding and induction to action.

Audiovisual educational materials	Understanding	Induction of action
1. What you should know about rheumatoid arthritis	“I can explain my illness to myself.” “Comply with the doctor’s recommendations, take my medicine and not forget”.	“Comply with the doctor’s recommendations, take my medicine and not forget”.
2. What you should know about your rheumatoid arthritis drugs	“If I take medicine, it relieves my pain, I’m able to work a little, otherwise I cannot work”. “That I comply with what the doctor says and that I do not suspend the medicine, thus healing and relieving the pain I have”.	“That I comply with what the doctor says and that I do not suspend the medicine, thus healing and relieving the pain I have”.
3. What are the instructions related to your medications?	“Tell the doctors where the pain is, if we have illnesses and not suspend the medicines”. “That I have to comply with the recommendations, such as taking the medicine and not mixing it, if I get more ill I must come here for a consultation”.	“That I do what the doctor says and that I do not mix the medicines”. “Yes, getting my medicine, that’s how we search for our life with medicines”.
4. Suggestions for controlling your rheumatoid arthritis symptoms	“Comply with what the doctors say”. “I place a cloth on my hand and the joints that are diseased”.	“If we talk to the doctors, tell them all about our illness, so they give us our medication so that we can get better”. “I place a cloth on my hand and when it calms down a bit, then I can do something”.
5. Drugs also expire!	“Do not put your medicine in the sun, it goes bad, I have to leave my medicine in a cool place”. “If the expiration date has passed, you should not take your medicine”.	“Take care of our medicines and see if we can still take them or if it is already off”.
6. Can your drugs cause discomfort?	“I should go if the medicine causes a negative reaction or if we do not use it correctly, we should go tell the doctor immediately”. “The body does accept some medications and there are some that it does not, you have to tell the doctors”.	“Tell doctors when the body does not accept a medicine or when it reacts negatively”. “He says not to forget my medicine and not to mix my medicines with herbs”.
7. Some doubts about rheumatoid arthritis	“I have to take care of myself, take my medication and I have to move my hands, exercise”. “We need to go to get check-ups, to the medical consultation, and those who are pregnant need to come to get check-ups and to the consultation”.	“When I’m well I can make my tortilla”.

A second version of the seven materials was generated at the end of this phase, with modifications addressing patients’ comments and concerns. Some of the modifications included: adding color to the drawings, indicating the area of pain, increasing the size of the drawings simulating the thermometer and lymphocytes, showing the passage of the drug through the esophagus and stomach, adding photographs of real tablets and capsules, adding sun rays to medicines, and adding symbols that indicate right and wrong. It is important to note that the photographs added were of people who were not wearing traditional Indigenous garments.

### 3.3 Phase 3: Quantitative validation

The second version of the seven audiovisual educational materials was quantitatively evaluated with 15 bilingual patients with RA [8]. The patients included were from the three main ethnic groups in Chiapas: Tzotziles (26.66%), Tzeltales (60%) and Ch’oles (13.3%). Table 2 shows the demographic characteristics of the patients. All patients were women with a mean age of 41 years; 93% of them had a maximum of 9 years of formal schooling, 87% were born in a rural area, but 80% now lived in an urban area. Regarding disease characteristics, 66% of participants had had RA for over 5 years. Additionally, all participants were under a combined scheme of DMARDs; only 20% of them were completely adherent. The reasons for non-adherence to DMARDs were forgetfulness (91.66%), suspending treatment because they felt good (41.66%), not following a schedule (41.66%), and discomfort related to DMARDs (58.33%).

**Table 2:** Characteristics of the participating patients during the quantitative evaluation.

Variables	n = 15
Age in years, mean (range)	41 (29–53)
Women n (%)	15(100)
Years of schooling n (%)	
<6	1 (6.7)
6–9	13 (86.6)
>9	1 (6.7)
Paid employment n (%)	8 (53.3)
Place of birth n (%)	
Rural	13 (86.6)
Urban	2 (13.4)
Place of residence n (%)	
Rural	3 (20)
Urban	12 (80)
Comorbidities n (%)	
Yes	1 (6.6%)
Years since onset of disease n (%)	
<5	5 (33.3)
5–10	6 (40)
>10	4 (26.7)
Morning stiffness n (%)	
Yes	7 (46.6)
Performs activities independently n (%)	
Yes	7 (46.6)
Medication adherence n (%)	
Yes	3 (20)

In terms of the specific goals of the audiovisual educational material (described in Table 3), an average score of 92.8% was achieved. Additionally, 62 total questions were asked during the interviews, most of them regarding audiovisual educational materials 6 and 7 which cover the discomfort that drugs and food can cause.

**Table 3:** Individual goals of the audiovisual educational materials

Audiovisual educative materials	Goals
1. What you should know about rheumatoid arthritis	•Clarify the main doubts about the disease. •Know the importance of following the instructions of the health professional.
2. What you should know about your rheumatoid arthritis drugs	•Know the two types of medicines used for your illness. •Know the benefits of using medications following the doctor’s instructions. •Know the reasons why changes are made in the indications. •Know the importance of not self-medicating.
3. What are the instructions related to your medications?	•Know the main indications that can be received from the health care team. •Know the importance of following the instructions of the health care team by identifying how the amount of medication prescribed and used impacts its effects. •Know the importance of maintaining good communication with the doctor and pharmacist. •Reaffirm the importance of not self-medicating.
4. Suggestions for controlling your rheumatoid arthritis symptoms	•Know that, to have better health results, in addition to following the rheumatologist’s indications, you can see other health professionals. • Know the main recommendations of some health professionals.
5. Drugs also expire!	•Know the importance of correctly preserving your medicines and what places you should avoid preserving them. •Understand the importance of not using improperly stored or expired medications.
6. Can your drugs cause discomfort?	•Know the main adverse drug reactions. •Know the importance of going to the doctor in case of an adverse drug reaction. •Know the importance of not self-medicating.
7. Some doubts about rheumatoid arthritis	•Know the reality regarding the main popular beliefs about the disease.

In terms of the efficacy components, Table 4 describes the scores obtained for each audiovisual educational material. Firstly, most of the images were well understood (82.8%) and thus the understanding score ranged from 73.3% to 100%. Secondly, both the attraction and the induction to action components of all the audiovisual educational materials obtained acceptable scores (88.3%–95% and 86.7%–100%, respectively, while the acceptance component reached the highest score of compliance (93.3% to 100%); two of the materials had 100% acceptance, while another two reached a score of 75%. Comparatively, the involvement component did not reach the required level, with scores ranging from 33% to 60%, with the exception of audiovisual educational material 6. These low scores were due to patients considering that the audiovisual educational materials were not directed at them, but at other patients.

**Table 4:** Individual quantitative evaluation of the five efficacy components.

Questions	Answers	Audiovisual educational materials n (%)						
		1	2	3	4	5	6	7
**Attraction**								
Was the video too short, too long, or an adequate length?	Alright/it takes little time	13 (86.7)	14(93.3)	14(93.3)	15 (100)	15(100)	13(86.7)	15 (100)
	More information is needed	2 (13.3)	1 (6.7)	1 (6.7)	0	0	2(13.3)	0
Did you like the video?	Yes	15 (100)	15 (100)	15 (100)	15 (100)	15 (100)	15 (100)	14(93.3)
	A bit	0	0	0	0	0	0	1 (6.7)
	No	0	0	0	0	0	0	0
Did you like the images?	Yes	14 (93.3)	13(86.7)	15(100)	14 (93.3)	14 (93.3)	14 (93.3)	14(93.3)
	Some	1 (6.7)	1 (6.7)	0	1 (6.7)	1 (6.7)	1 (6.7)	1 (6.7)
	No	0	1 (6.7)	0	0	0	0	0
Did you like the colors?	Yes	11 (73.3)	11 (73.3)	13 (86.7)	13 (86.7)	12 (80)	11 (73.3)	13(86.7)
	It lacks color	2(13.3)	3(20)	2(13.3)	1(6.7)	2(13.3)	3(20)	1(6.7)
	No	2(13.3)	1(6.7)	0	1(6.7)	1(6.7)	1(6.7)	1(6.7)
	**n** **=** **60 (%)**	**53(88.3)**	**53(88.3)**	**57 (95)**	**57 (95)**	**56 (93.3)**	**53 (88.3)**	**56 (93.3)**

**Comprehension**								
Did you understand the information in the video?	Yes	11 (73.3)	14 (93.3)	14 (93.3)	11 (73.3)	14 (93.3)	15 (100)	13(86.7)
	A bit	0	1 (6.7)	1 (6.7)	4 (26.7)	1 (6.7)	0	1 (6.7)
	No	4 (26.7)	0	0	0	0	0	1 (6.7)
	**n** **=** **15 (%)**	**11 (73.3)**	**14 (93.3)**	**14 (93.3)**	**11 (73.3)**	**14 (93.3)**	**15 (100)**	**13 (86.7)**

**Acceptance**								
Is there a word or image that makes you feel upset, offended or angry?	No	15 (100)	14 (93.3)	15 (100)	15 (100)	15 (100)	15 (100)	14(93.3)
	Yes	0	1 (6.7)	0	0	0	0	1 (6.7)
	**n** **=** **15 (%)**	**15 (100)**	**14 (93.3)**	**15(100)**	**15 (100)**	**15 (100)**	**15 (100)**	**14 (93.3)**

**Involvement**								
Who do you think this video is for?	It involves other patients	15 (100)	15 (100)	15 (100)	15 (100)	15 (100)	15 (100)	15 (100)
	**n** **=** **15 (%)**	15 (100)	15 (100)	15 (100)	15 (100)	15 (100)	15 (100)	15 (100)
	It involves the participating patients	8 (53.3)	5 (33.3)	8 (53.3)	9 (60)	9 (60)	11 (73.3)	8 (53.3)
	**n** **=** **15 (%)**	**8 (53.3)**	**5 (33.3)**	**8 (53.3)**	**9(60)**	**9(60)**	**11 (73.3)**	**8 (53.3)**

**Induction to action**								
Is this video asking you to do something?	Yes	15 (100)	14 (93.3)	14 (93.3)	14 (93.3)	15 (100)	15 (100)	13 (86.7)
	No	0	1 (6.7)	1 (6.7)	1 (6.7)	0	0	2(13.3)
	**n** **=** **15 (%)**	**15 (100)**	**14 (93.3)**	**14 (93.3)**	**14 (93.3)**	**15 (100)**	**15 (100)**	**13 (86.7)**

A third version of the audiovisual educational materials was created based on the above results, considering the concerns expressed by the patients, as well as their social environment. Therefore, elements related to the Indigenous population and its environment were included, such as: photographs of the Esquipulas Clinic and daily life activities at home; characters with a younger appearance and traditional Indigenous garments; and symbols characteristic of the community such as braids with colorful ribbons and embroideries. Around 40 photographs were added in total, related to sadness, joy, activities, and pregnancy, among others.

The involvement component was reevaluated after said modifications were made. A further ten patients aided in this reevaluation, nine of whom were bilingual and one monolingual, with a mean age of 39 years. As a result of this evaluation, the seven audiovisual educational materials reached the maximum score (100%), suggesting that the photographs helped patients identify themselves as the intended audience.

## 4 Discussion and conclusion

### 4.1 Discussion

Audiovisual educational materials aimed at patients with RA should be a pillar to achieve treatment efficacy [23], since their use has been shown to improve patient participation in their health care, as well as therapeutic adherence [9,[24], [25]]. However, there is scarce development of educational strategies adapted and validated for patients, according to their cultural and sociodemographic context [[26], [27]]. Therefore, the need to have material that involves the patients in their health care is evident.

This study resulted in the design and validation of seven culturally appropriate audiovisual educational materials for patients with RA from the highlands of Chiapas. The process of validation demonstrates the importance of cross-cultural adaptation of audiovisual material to achieve high levels of compliance with efficacy components. Since the cultural identity and worldview of different ethnic groups is rarely considered in educational materials [28], cross-cultural adaptation should be carried out gradually, incorporating elements of cultural identification with each improved version of the audiovisual material (Fig. 1).

As described in the previous study [8], according to participating specialists and patients, the audiovisual materials address seven topics that were considered pertinent and sufficient for a patient with RA to identify the symptoms of their disease, the proper use of medications, the consequences of improper use, and concerns or myths about their disease or medications. In this sense, this audiovisual material can be used as a complement to a pharmaceutical care program [29] allowing an increase in shared decision-making between patients with RA and rheumatologists, as well as encouraging patients to make better health care decisions.

As described in previous studies [19,30], the cross-culturally adapted instrument must be validated to determine whether the material complies with the efficacy components and the objectives for which it was designed. In this sense, a strength of our study is precisely that the cross-cultural adaptation was followed by a validation process.

The qualitative validation phase allowed patients to share their perspectives on the audiovisual educational material and some cultural elements, leading to modifications and improvements. In this phase we found that the audiovisual educational material drew attention and is pleasant (complies with attraction), does not have elements that generate anger, annoyance or offense (complies with acceptance), and has recommendations that are easy for Tzotzil patients to identify and carry out (complies with induction to action). However, the audiovisual educational material did not comply with understanding or involvement, which may hinder patient empowerment and ability to achieve clinical remission. This lack of compliance can be attributed to the lack of color in the drawings leading to in understanding, and the lack of characteristic elements of the culture leading to non-involvement. These results coincide with what was observed in the study by Velazco [31], where an addition of color was requested for audiovisual educational materials to comply with the efficacy components. Finally, the audiovisual educational materials were improved by adding photographs of relevant cultural elements, identified as diet, daily activities, and the use of herbs, which are characteristic of the population.

Given that the original audiovisual educational material, previously validated in a non-Indigenous population [8], complied with the efficacy components with values greater than 86.6%, we erroneously assumed that similar results would be obtained in the first quantitative validation with Indigenous patients. However, we obtained values greater than 53.3% which, according to UNICEF [10], are not acceptable and indicate that the audiovisual educational material is not culturally suitable for the participating Indigenous population. Since the differences in results were likely due to the differences in populations, the cross-cultural adaptation (inclusion of images and cultural references of Indigenous patients) was able to increase efficacy to values greater than 73.3% and a mean of 94.51% ± 4.98%.

During the quantitative phase, we observed high fulfillment of the objectives of the audiovisual educational material (92.85%), as well as of the components of attraction (>88.3%), understanding (>73.3%), induction to action (>86.7%), and acceptance (>93.3%). However, the involvement component (53.3%) was not met in the first quantitative validation in six of the seven audiovisual educational materials (1–5 and 7), because the participants considered that the audiovisual educational material was targeted, not at themselves, but at other patients with RA. This differs from our previous study [8], in which compliance with involvement was 83.9%. Importantly, this lack of involvement can negatively affect the follow-up of the recommendations given and thus impact overall health status of the target population [10]. In general, we observed that the lack of culturally sensitive elements in the audiovisual educational materials causes Indigenous patients to not feel identified with them. This is confirmed by the fact that the only audiovisual educational material that met the involvement component in the first quantitative validation contained photographs of the drugs that patients receive; this is a component of their environment that made patients feel that this audiovisual educational material was made for them. Indeed, participants commented that the audiovisual educational materials were intended for other patients, since the people shown in them did not present characteristics of the Indigenous culture.

In accordance with what has been reported in previous studies [[9], [14], [15], [30]], replacing the drawings with photographs and colors with socio-cultural representation to generate an environment close to their reality favors patient involvement. In the present study, the incorporation of traditional garments, as well as images that illustrated activities of daily life, significantly improved the involvement component, increasing from 53.3% to 100%. These results also coincide with a previous study [31] showing that Indigenous populations are visual learners.

Unlike a previous study with the Misak Indigenous population in Colombia [32], in the present study we observed that the participants needed to ask questions to affirm the information received. This fact confirms that the use of educational material, by itself, is limited, and requires a health professional to complete the educational process. In that sense the pharmacist can play a relevant role in promoting communication with the patient and, in this case, improving their health care. In our study, the fact that the pharmacist had participated from the stage of the identification of the topics and the design of the audiovisual educational material according to the perspective and information needs of the patients with RA [8], meant that it was not necessary to modify the topics for the new versions even if the presentation could be improved, since the material already contained the necessary content for participating Indigenous patients even if the presentation could improve.

This study has some limitations, such as the fact that it is a monocentric study, and the audiovisual educational material was only translated into one Indigenous language out of the 14 presents in the state [33]. Likewise, there are limitations regarding the representativeness of the participants, since all were women, mostly urban residents (80%), and regularly attended their medical consultations. However, our participants represented three Indigenous ethnicities (Tzotzil, Tzeltal, and Ch’ol), as well as a wide range of different ages, disease duration, and adherence; thus, it remained a diverse group. However, we propose that future research include male patients and patients who have chosen not to attend medical consultations, to provide a broader perspective of the Indigenous population.

### 4.2 Innovation

The main innovation of the present study is the translation and adaptation of educational materials from Spanish into Tzotzil Indigenous language and culture; this is essential to ensure that the materials are effective in meeting their specific educational goals and enhancing learning for these patients.

The cross-cultural adaptation and validation of an audiovisual material is enough to improve its compliance with efficacy components among patients from culturally different communities, reaching high levels of compliance with educational goals. The key aspect of these materials for patients is not the graphic design or the quality of the photographs, but their efficacy. Previous studies have confirmed that very few freely accessible audiovisual materials meet the efficacy components. When the audiovisual educational materials are intended to be used for patients with different linguistic and cultural references, cross-cultural adaptation is essential to ensure that they can meet said educational purposes. These results suggest that cross-cultural adaptation and validation should be carried out for each new culturally different group of patients.

### 4.3 Conclusion

The cross-cultural adaptation and validation of seven audiovisual educational materials for Indigenous patients with RA successfully complied with the efficacy components, making the material culturally appropriate for these patients. This process involved careful adjustments to ensure that the content was not only linguistically accurate, but also met cultural sensitivity. Furthermore, the participation of a multidisciplinary team, including pharmacists and bilingual translators, played a crucial role in the process, as it reduced knowledge, cultural and linguistic gaps.

This study provides a replicable model for developing patient-centered educational materials that could be applied to other vulnerable populations with unique cultural and linguistic needs.

## Funding

The production and validation of the audiovisual educational materials were supported by an ILAR Project Funding grant 2019 (J.-L.M.-H.).

## CRediT authorship contribution statement

**Joana Aguilar-Castillo:** Writing – review & editing, Writing – original draft, Visualization, Validation, Supervision, Software, Resources, Project administration, Methodology, Investigation, Formal analysis, Data curation, Conceptualization. **Ingris Peláez-Ballestas:** Writing – review & editing, Supervision, Project administration, Methodology, Conceptualization. **José-Luis Montiel-Hernández:** Writing – review & editing, Supervision, Project administration, Funding acquisition, Conceptualization. **Cairo Toledano-Jaimes:** Writing – review & editing. **Mario-Alberto Garza-Elizondo:** Writing – review & editing. **David Zepeda-González:** Writing – review & editing, Resources. **Diana-Lizbeth Gómez-Galicia:** Writing – review & editing, Writing – original draft, Supervision, Project administration, Methodology, Investigation, Conceptualization.

## Declaration of competing interest

The authors report no competing interests.
